# High-Performance PEEK/MWCNT Nanocomposites: Combining Enhanced Electrical Conductivity and Nanotube Dispersion

**DOI:** 10.3390/polym16050583

**Published:** 2024-02-21

**Authors:** Sofia Silva, José M. Barbosa, João D. Sousa, Maria C. Paiva, Paulo F. Teixeira

**Affiliations:** 1 CeNTI—Centre for Nanotechnology and Smart Materials, R. Fernando Mesquita 2785, 4760-034 Vila Nova de Famalicão, Portugal; jbarbosa@centi.pt (J.M.B.); jdsousa@centi.pt (J.D.S.); pteixeira@centi.pt (P.F.T.); 2 Department of Polymer Engineering, Institute for Polymers and Composites, University of Minho, 4800-058 Guimarães, Portugal

**Keywords:** polyether ether ketone, carbon nanotubes, nanocomposites, melt extrusion parameters, electrical conductivity, MWCNT dispersion

## Abstract

High-performance engineering thermoplastics offer lightweight and excellent mechanical performance in a wide temperature range. Their composites with carbon nanotubes are expected to enhance mechanical performance, while providing thermal and electrical conductivity. These are interesting attributes that may endow additional functionalities to the nanocomposites. The present work investigates the optimal conditions to prepare polyether ether ketone (PEEK)/multi-walled carbon nanotube (MWCNT) nanocomposites, minimizing the MWCNT agglomerate size while maximizing the nanocomposite electrical conductivity. The aim is to achieve PEEK/MWCNT nanocomposites that are suitable for melt-spinning of electrically conductive multifilament’s. Nanocomposites were prepared with compositions ranging from 0.5 to 7 wt.% MWCNT, showing an electrical percolation threshold between 1 and 2 wt.% MWCNT (10^7^–10^2^ S/cm) and a rheological percolation in the same range (1 to 2 wt.% MWCNT), confirming the formation of an MWCNT network in the nanocomposite. Considering the large drop in electrical conductivity typically observed during melt-spinning and the drawing of filaments, the composition PEEK/5 wt.% MWCNT was selected for further investigation. The effect of the melt extrusion parameters, namely screw speed, temperature, and throughput, was studied by evaluating the morphology of MWCNT agglomerates, the nanocomposite rheology, and electrical properties. It was observed that the combination of the higher values of screw speed and temperature profile leads to the smaller number of MWCNT agglomerates with smaller size, albeit at a slightly lower electrical conductivity. Generally, all processing conditions tested yielded nanocomposites with electrical conductivity in the range of 0.50–0.85 S/cm. The nanocomposite processed at higher temperature and screw speed presented the lowest value of elastic modulus, perhaps owing to higher matrix degradation and lower connectivity between the agglomerates. From all the process parameters studied, the screw speed was identified to have the higher impact on nanocomposite properties.

## 1. Introduction

High-temperature engineering thermoplastics are gaining growing interest as they provide a lightweight solution, with outstanding properties, at high temperatures [1,2,3]. Polymers that can fulfil these requirements are grouped together as high-temperature polymers. Polyether ether ketone (PEEK), poly aryl ether ketone (PAEK), polyetherimide (PEI), and polyether sulphone (PES) are members of this group [3,4,5,6]. PEEK is a semi-crystalline thermoplastic and has been widely used as the matrix for reinforced plastics and nanocomposites due to its extraordinary properties of excellent thermal stability and chemical inertness, excellent strength and stiffness, good heat resistance, outstanding self-lubricating properties, flame retardancy, physical structural stability, high insulation, and service temperature up to 300 °C [2,5,7,8,9,10]. The drawbacks associated with PEEK include its elevated cost and high processing temperature (attributed to its exceptionally high melting point and increased melt viscosity) [11]. Furthermore, PEEK is characterized by low electrical conductivity, limiting its applicability in certain applications, such as space, submarine, road transport vehicles, building materials, battery components, electrodes, electromagnetic interference (EMI) shields, structural health monitoring, electronic devices, and conductors [1,3,5,12,13,14,15].

To overcome this limitation, various nanocarbons can be incorporated into the PEEK matrix. Carbon nanotubes (CNTs) are widely used owing to their exceptional mechanical properties, which are combined with a high aspect ratio and nanometric dimensions, electrical and thermal conductivities, low density, high surface area, and corrosion resistance [1,16,17]. However, the transfer of properties from the matrix to the CNTs is challenging, as they tend to form entangled CNT agglomerates that are stabilized by physical interactions such as Van der Waals forces. Agglomerates can act as imperfections, reducing mechanical toughness and the number of nanotubes available for electrical percolation [15,18,19]. Thus, it is important to reduce the percolation threshold of CNT-reinforced composites, optimizing the CNT volume fraction required to achieve the target properties, while attaining good dispersion of the CNT agglomerates and reducing the composite price.

Thermoplastic composites usually present an electrical percolation threshold at around 0.05–5 wt.% CNT [15,20,21,22,23]. PEEK–CNT composites produced via various techniques have been reported to present a percolation threshold in the range of 1 to 5 wt.% CNT, in applications such as high-performance EMI shielding and electrostatic discharge materials for advanced technologies [22]. Although there are many publications dealing with the electrical percolation threshold for polymer/CNT composites [12,16,18,24,25] produced by melt processing, only a limited number of publications report on PEEK/CNT composites [20,21,26,27,28,29].

The electrical, thermal, and mechanical properties of the nanocomposites are affected by the type of CNT used, as well as by the polymer matrix, processing method, and conditions adopted [1,16,17,19,30]. Irrespective of the processing method selected, the process parameters need to be optimized to achieve electrical conductivity at the lowest possible CNT loading, while enhancing or at least not significantly affecting the mechanical behaviour [16]. Melt processing of thermoplastic-based nanocomposites is the favoured route to produce electrically conductive or electrostatic dissipative polymer composites containing carbon nanotubes [18]. In order to optimize melt-compounding by extrusion and achieve optimum CNT dispersion, the process parameters that require optimization are typically the screw configuration, screw speed, throughput/residence time, and temperature profile [31]. During melt-mixing, varying screw speed changes the shear rate applied to the polymer melt in the twin-screw extruder. It was reported by several authors [16,18,31,32,33] that dispersion improves with increasing screw speed due to the associated higher stress levels generated, applied by the melt on the CNT agglomerates, and helps promote agglomerate breakup. However, compositions below and above the percolation threshold respond differently to these effects [32]. High shear applied to the polymer melt may result in CNT breaking, and hence to a reduction in their length [31]. Pötschke et al. [32] reported a significant effect of screw speed on the dispersion of MWCNT, with a different result for compositions below and above the percolation threshold. Also, the authors found that viscous dissipation could induce degradation, leading to deterioration of the composite performance [18]. Conversely, lower screw speeds impart lower shear and kneading forces and favour dispersion through the erosion mechanism [25]. Therefore, a compromise must be reached to avoid the extreme effects of too low or too high screw rotational speeds [18,25]. The processing temperature during mixing mainly influences the viscosity of the polymer. At lower melt viscosity, higher polymer chain mobility enables a better and faster wetting of CNT as well as a faster infiltration of CNT agglomerates with polymer [12]. Conversely, higher viscosity may generate the higher hydrodynamic stresses required for agglomerate rupture [34]. Besides these parameters, increasing the throughput causes reduction in the residence time and an increase in the degree of screw fill, both hindering CNT dispersion [18]. The knowledge gained about the influence of processing conditions on composite properties, reported for different polymers [12,16,18,24,25,33,35,36,37], is quite relevant; however, only a limited number of publications report the melt processing of PEEK/CNT composites [15,24]. In particular, the literature lacks research about the preparation of these composites, aiming at the production of PEEK/CNT fibres by melt-spinning technology. This technology presents specific requirements; while the formation of electrically conductive composites benefits from good CNT agglomerate dispersion, melt-spinning of multifilament requires the absence of CNT agglomerates with sizes above a given threshold, typically with an equivalent diameter within 1–10 μm [38,39], which may jeopardize the fibre spinning process.

During the melt-spinning process, increasing the stretch increases the orientation of the macromolecules and CNT along the yarns [40]. As the orientation progresses, the density of CNT contacts in the CNT network decreases, resulting in the interruption of the percolation path [41]. Despite improving the mechanical properties of the yarns, the increase in orientation usually leads to a decrease in electrical conductivity [42]. Notably, there is a consensus that achieving a uniform dispersion of CNTs is essential for maximizing mechanical reinforcement. However, it was observed that the presence of a number of agglomerates is necessary for establishing an efficient conductive network [43]. Marischal et al. [41] produced carbon black (CB) composites with the objective of studying the influence of the melt-spinning process parameters on the electrical properties of the multifilament. After extrusion of composites with 11 wt.% CB, the electrical conductivity of 10^1^ S/m dropped sharply to 10^−6^ S/m in the multifilament. Similarly, Bouchard et al. [42] analysed the differences in electrical properties arising from the composite melt extrusion and the production of multifilament’s by melt-spinning of poly(hydroxy ether) of bisphenol A with CNT. The electrical conductivity of the multifilament produced with the 1.5 wt.% CNTs composite decreased from 4.50^−1^ to 3.55^−11^ S/m, compared to composites produced by melt extrusion. Thus, the multifilament has not yet reached the threshold of electrical percolation, and it is necessary to increase the CNT content and/or promote dispersion. The goal of producing an electrically conductive multifilament based on polymer/CNT composites has not been reached, requiring further investigation of the balance between CNT concentration, dispersion, and processing parameters that may enable the increase in multifilament electrical conductivity.

According to the literature, the correlation between the electrical properties and morphology of PEEK/CNT nanocomposites is still little investigated. Therefore, in this study, the electrical percolation threshold of PEEK/CNT nanocomposites was investigated using melt-compounding by twin-screw extrusion. A detailed study was carried out to correlate the morphology and electrical conductivity of the composites. The influence of the extrusion processing parameters (screw speed, profile temperature, and throughput) on the composite’s electrical conductivity and the MWCNT dispersion was assessed. To evaluate these properties, electrical conductivity, optical and scanning electron microscopy, and thermal and rheologic properties were investigated. The main objective was to produce PEEK/CNT composites with adequate properties for further processing using melt-spinning technology.

## 2. Materials and Methods

### 2.1. Materials

Two PEEK grades were used in this study, namely (i) PEEK KetaSpire KT-880 NT (neat PEEK) form Solvay (Brussels, Belgium), with 0.01 wt.% calcium stearate lubricant, a semi-crystalline thermoplastic with a glass transition temperature (T_g_) of 147 °C, melting temperature (T_m_) of 343 °C, melt flow index of 36 g/10 min (at 400 °C; 2.16 kg), and a density of 1.3 g/cm^3^; and (ii) a PEEK masterbatch, PLASTICYL^TM^ PEEK1001, with 10 wt.% of MWCNTs, supplied by Nanocyl (Sambreville, Belgium). PLASTICYL^TM^ PEEK1001 (PEEK/MWCNT masterbatch) presents a density of 1.274 g/cm^3^, melt flow index of 1.4 g/10 min (at 400 °C; 2.16 kg; 4 wt.% MWCNTs), volume resistivity of 10 Ω·cm (at 4 wt.% MWCNTs), and surface resistivity of 10^3^ Ω·sq. (measured at 4 wt.% MWCNTs). The nanotubes have a typical diameter of 9.5 nm and an average length of 1.5 μm. The density of MWCNT is approximately 1.66 g/cm^3^ [18]. PEEK/MWCNT masterbatch with 10 wt.% MWCNT was used to form PEEK nanocomposites with lower MWCNT content. The use of masterbatch dilution was selected for safe handling of the MWCNTs. 

### 2.2. Preparation of PEEK/MWCNT Nanocomposites

#### 2.2.1. Melt-Compounding

**Figure 1:**
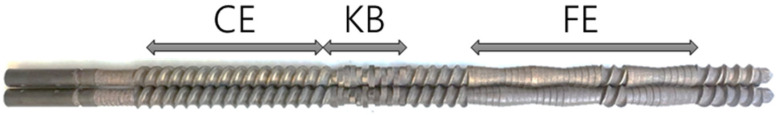
Screw profile, including conveying (CE), kneading (KB), and folding (FE, chaotic mixing) used to produce PEEK/MWCNT nanocomposites.

The nanocomposites were manufactured using an extrusion line from Rondol Technology Ltd., Model LabTwin 21 (Nancy, France). This equipment is a 21 mm intermeshing co-rotating twin-screw benchtop extruder with a length to diameter ratio (L/D) of 25. The barrel of the twin-screw extruder is equipped with four heating zones. Before extrusion, all materials were dried in an indirect heated dehumidifying drying oven at 150 °C for 4 h. The dried neat PEEK and PEEK/MWCNT masterbatch were hand-mixed and added to the hopper at the main feeding zone, using a gravimetric feeder. The extrudate strand, approximately 2 mm in diameter, was cooled in a water bath and then pelletized into 2 mm pellets. The screw configuration was designed using conveying (CE), kneading (KB), and folding (FE) elements. The initial part of the screw consists of a conveying section, followed by an intensive mixing section that includes kneading elements (5 KB 30°, 3 KB 60°, and 4 KB 90°). Subsequently, there is a chaotic mixing zone constituted by a folding element. The chaotic mixture characteristics were set to increase the pressure and the residence time in the melting and mixing zone. Figure 1 shows the screw profile used for the PEEK/MWCNT nanocomposites production. 

#### 2.2.2. Experimental Planning

In the first set of compounding experiments, PEEK/MWCNT nanocomposites containing 0.5, 1, 2, 3, 4, 5, and 7 wt.% MWCNT were prepared to investigate the electrical percolation threshold, as well as the electrical conductivity achieved above the percolation. Selected extrusion parameters, based on technical data sheets and the literature, were a flat temperature profile of 365 °C, throughput of 3 kg/h, and screw speed of 175 RPM.

In the second set of compounding experiments, the influence of the extrusion processing parameters on the nanocomposite’s electrical conductivity and MWCNT dispersion were investigated. The most relevant processing parameters reported in the literature [37], namely, profile temperature, throughput, and screw speed, were varied. Two levels, designated as “low” and “high”, were tested as indicated in Table 1, leading to eight extrusion processing experiments. 

### 2.3. Characterization Methods

The state of MWCNT macrodispersion in the composites was studied using light transmission microscopy (LM). Thin nanocomposite slices with a thickness of 4 μm was cut perpendicular to the direction of extrusion on at least four different strands at room temperature using an ultramicrotome (Leica EM UC6) equipped with a glass knife. Slices were cut from different regions of the same nanocomposite filament to ensure the statistical significance. Images were acquired using a Leica DM2500 M transmission microscope, combined with a digital camera Leica K3 C, analysed with the software Leica LAS X (version 5.1.0.25593). A minimum of 4 mm^2^ micrograph area was investigated for each sample. The agglomerate area ratio A/A0 was calculated from the optical micrographs as the ratio of the area “A”—sum of the areas of all the agglomerates present—to the total area of the micrographs analysed, “A0”. This ratio was calculated in percentage. For each slice, the agglomerate area ratio of the nanocomposites was evaluated according to the following Equation (1) [44,45],

(1) $$Agglomerate\;area\;ratio=\frac{A}{A0}\times 100$$

Image analysis was performed using Adobe Photoshop (version 25.4.0) and Image J software (version 1.53t). Grayscale thresholding was used to target the foreground agglomerates from the background matrix, and particle analysis provided the area of each agglomerate. The well-dispersed nanoparticles cannot be observed by optical microscopy, and the areas of the MWCNT agglomerates greater than 5 µm^2^ were measured (according to ISO 18553 standards [46]). The distribution of the agglomerate areas was organized in classes of 250 μm^2^ and was presented to include all the agglomerate sizes (typically from 5 to 8000 µm^2^).

To assess the state of dispersion and interface between MWCNTs and the PEEK matrix, scanning electron microscopy (SEM) was performed on a NanoSEM—FEI Nova 200 microscope. The nanocomposites analysed by SEM were prepared by cryogenic fracture of the extruded strands and sputtering of the cryo-fractured surface with palladium-gold (80–20%).

Electrical resistivity measurements of PEEK/MWCNT nanocomposites were carried out on at least 10 samples of the extruded strand of each composition, cut with 15 mm length, with a razor blade. Electrical contacts made of conductive silver paint ((CI-1036 Silver Conductive Ink)) were cured on both ends of the specimens. In order to measure the volume resistivity in the wide range from insulator to electrically conductive, two measuring devices were employed. Low-resistivity measurements (<10^6^ ohm·cm) were conducted by a two-probe measurement using a Fluke 279 FC Digital Multimeter from Fluke Corporation (Everett, WA, USA), while high volume resistivities (>10^6^ ohm·cm) were measured using a Picoammeter Keithley 6487 resistance from Keithley Instruments, Inc. (Cleveland, OH, USA), meter as two-probe measurements.

Volume resistivity was calculated according to Equation (2), where ρ is the volume resistivity in (Ω·cm), *R* is the measured electrical resistance (Ω), *A* is the sample cross-section (cm^2^), and *d* is the distance between electrodes (cm).

(2) $$\rho =\frac{R\times \;A}{d}$$

Thermogravimetric analysis (TGA) was conducted to assess the thermal stability of PEEK/MWCNT nanocomposites, using a TA-Q500 thermobalance (SDT Q600 V20.9 Build 20) from TA Instruments (New Castle, DE, USA). Samples of ~20 mg were heated at a heating rate of 10 °C/min, from 40 to 950 °C, with a gas purge of 100 mL/min. The analysis was performed under nitrogen atmosphere. At least two runs were carried out per sample. 

Dynamic rheological measurements were conducted using an HR 10 rotational rheometer from TA Instruments (New Castle, DE, USA) fitted with a steel parallel-plate geometry (25 mm diameter and 1.5 mm gap). The linear viscoelastic response of the samples was measured with oscillatory frequency sweeps over an angular frequency (ω) range of 0.025 to 100 Hz, carried out at 400 °C under a constant strain amplitude of 1%. Prior to testing, the samples were oven-dried at 150 °C for 4 h.

## 3. Results and Discussion 

### 3.1. Electrical Percolation Threshold of the Nanocomposites

The electrical percolation threshold of the PEEK/MWCNT nanocomposites is observed at the MWCNT concentration that raises the composite electrical conductivity by several orders of magnitude. The MWCNT concentration at which the increase in the conductivity is observed is influenced by several factors, the filler dispersion being a key element [47].

#### 3.1.1. Electrical Conductivity

**Figure 2:**
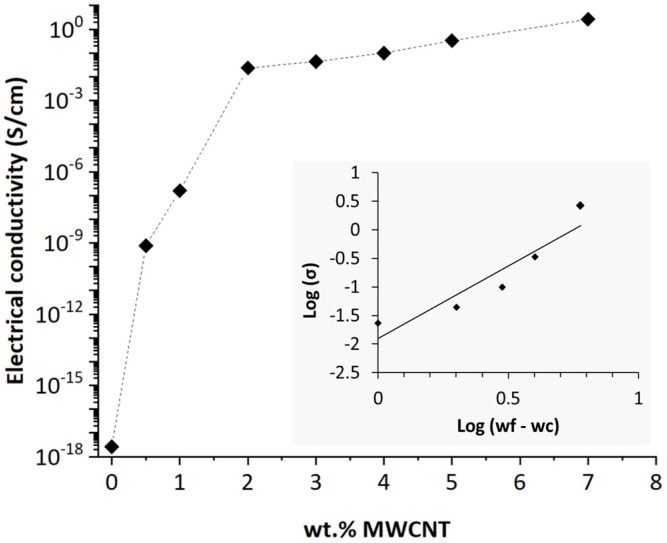
Electrical conductivity of PEEK/MWCNT nanocomposites with increasing MWCNT content. The inset shows the plot of the optimal fitting of the *log σ* vs. *log* (*w_f_* − *w_c_*).

The electrical conductivity (σ) of PEEK/MWCNT nanocomposites up to 7 wt.% of filler content are shown in Figure 2. The value for zero concentration corresponds to the conductivity of neat PEEK (2.63 × 10^−18^ S/cm). As the amount of MWCNT increases from 0.5 to 2 wt.% MWCNT, the electrical conductivity increases fast. From 1 to 2 wt.%, a percolation threshold is reached with electrical conductivity varying from 1.59 × 10^−7^ to 2.34 × 10^−2^ S/cm. An interconnected network of MWCNT, comprising individual MWCNTs and agglomerates, was formed at this loading [16,22,26].

The electrical percolation threshold measured for PEEK/MWCNT nanocomposites in Figure 2 is within the range reported in the literature for PEEK nanocomposites [15,20,21,22,23,24,27,28,29]; an electrical percolation threshold between 1 and 3 wt.% of MWCNT is typically reported. With the incorporation of 1 to 4 wt.% MWCNT in the PEEK nanocomposites, the electrical conductivity levels attained allow electrostatic discharge applications, applications in the semiconductor range and, for the nanocomposites produced with >4 wt.% MWCNT, shielding applications (>10^−2^ S/cm) [13,18,48].

The classical percolation theory [49] estimates the dependence of electrical conductivity on filler concentration beyond the electrical percolation threshold based on a power law as represented in Equation (3), where $\sigma_{0}$ is the conductivity of the filler, $w_{f}$ is the filler weight fraction, $w_{c}$ is the critical weight fraction at the electrical percolation threshold, and t is the critical exponent, related to network dimensional aspects.

(3) $$\sigma =\sigma_{0}{\left(w_{f}-w_{c}\right)}^{t}$$

Equation (3) is valid only at the concentration above the percolation threshold, that is, when $w_{f}$ > $w_{c}$. To estimate the percolation threshold based on the experimental results of electrical conductivity vs. wt.% MWCNT, the experimental curve was analysed based on the double logarithmic relation: *log* σ vs. *log* ($w_{f}$ − $w_{c}$), (see the inset in Figure 2) varying the value of $w_{c}$ until the best linear fit was obtained. The $w_{c}$ value thus obtained was 1 wt.% of MWCNT (for a coefficient of correlation, R^2^, of 0.87), which is in accordance with the studies discussed. For MWCNT–polymer composites, the range of critical exponent values fitted from experimental measurements found for different systems indicates that the t value is not universal. Bauhofer and Kovacs [23] indicated t values obtained from experiments for three-dimensional percolating systems between 1.3 and 4. In this study, the estimated value of t was 2.47, providing evidence for a three-dimensional conductive network of MWCNT within the polymer matrix.

#### 3.1.2. Rheological Properties

Determining the rheological percolation threshold is also crucial since the rheological behaviour of these composites are, generally, significantly different below and above the percolation threshold, affecting parameters such as viscosity and consequently, processability [21,50].

**Figure 3:**
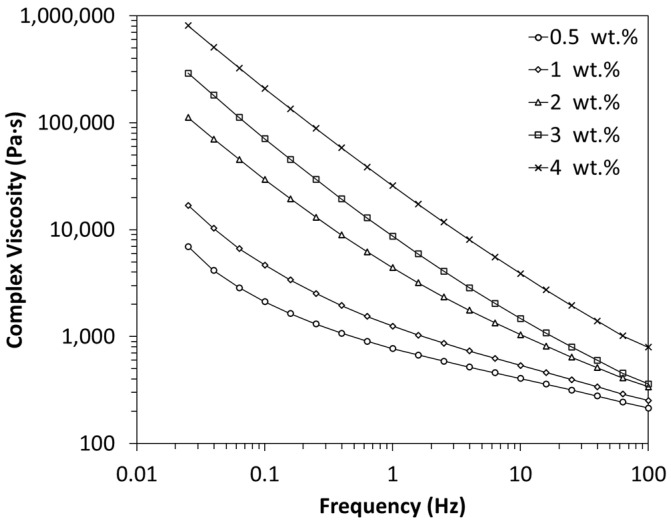
Complex viscosity of PEEK/MWCNT nanocomposites as a function of frequency at 400 °C.

**Figure 4:**
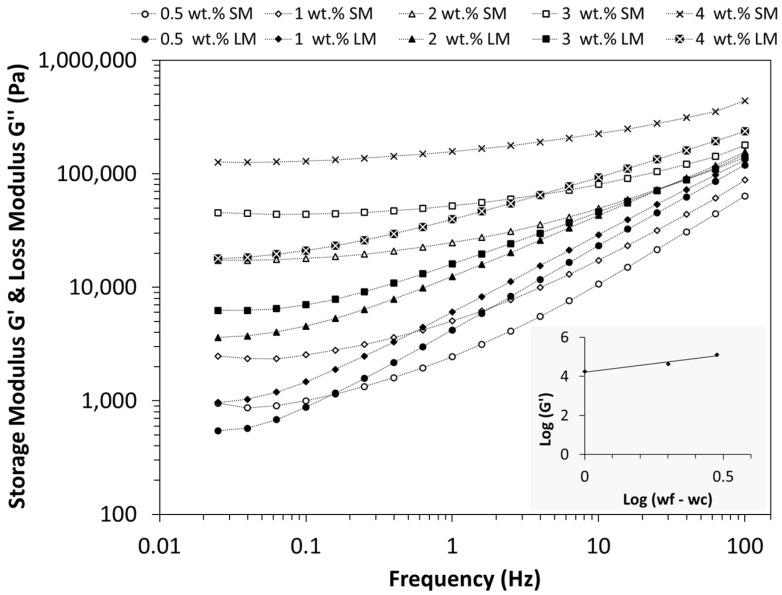
Storage modulus, *G*′ (empty symbols) and loss modulus, *G*″ (filled symbols) of the PEEK/MWCNT nanocomposites as a function of frequency at 400 °C. The inset shows the optimal fitting of the *log G*′ vs. *log* (*w_f_* − *w_c_*) plot.

The effect of the MWCNT concentration on the linear viscoelastic response of the nanocomposites at 400 °C is shown in Figure 3 and Figure 4. The frequency-dependent behaviour of the dynamic complex viscosity (η*) is depicted in Figure 3, while Figure 4 illustrates the storage and loss modulus (*G*′ and *G*″) against frequency (Hz), covering MWCNT contents ranging from 0.5 to 4 wt.% for PEEK/MWCNT nanocomposites. Figure 3 shows that PEEK/MWCNT composites demonstrate plastic behaviour of complex viscosity versus frequency in the whole concentration range studied. The addition of MWCNTs significantly alters the flow behaviour of the PEEK/MWCNT nanocomposites. The viscosity gradually increases as the MWCNT content increases. This effect is more pronounced at the low-frequency region, which is related to the increase in particle–polymer interactions and the reduction in flowability of the PEEK chains. A change in the shape of the viscosity–frequency curve is observed, from 1 to 2 wt.% MWCNT loading. The viscosity curves for nanocomposites containing >1 wt.% exhibit a much steeper slope at the frequency region, with the nanocomposite containing a higher filler concentration demonstrating a pronounced shear-thinning effect. This range (1–2 wt.%) can be regarded as a rheological percolation threshold and is in good agreement with the electrical conductivity data.

Figure 4 depicts experimental results for the storage modulus and the loss modulus as functions of frequency at 400 °C. Both *G*′ and *G*″ exhibit an increase with MWCNT concentration. For all concentrations, *G*′ and *G*″ reach a plateau at low frequencies, suggesting that the percolated MWCNTs may form a pseudo-solid-like network with strong interactions between the polymer and particles [51]. However, the composites with the filler content of 0.5 and 1 wt.% show a liquid-like flow behaviour with a typical transition from elastic to viscous behaviour (*G*″ > *G*′) at a critical frequency of 0.15 and 0.53 Hz, respectively. PEEK/MWCNT nanocomposites with MWCNT > 2 wt.% show a predominantly elastic response (*G*′ > *G*″, plateau in *G*′) over the entire frequency range. This may be associated with interconnection between nanotubes, resulting in percolation. The rheological percolation threshold is usually associated with the structural liquid-to-solid transition, indicating the formation of a percolation network of interconnected nanoparticles, immobilized with matrix polymer [52,53]. Different approaches are used in the literature to determine the percolation threshold [54].

In line with the methodology employed for electrical conductivity, the classical percolation theory was also applied to estimate the rheological percolation threshold, using Equation (4):

(4) $$G^{\prime }\propto {\left(w_{f}-w_{c}\right)}^{t}$$

A double logarithmic plot of *log G*′ (at frequency = 0.1 Hz) vs. *log* (*w_f_* − *w_c_*) was generated (refer to the inset in Figure 4). The value of *w_c_* was systematically varied until the optimal linear fit was achieved, resulting in a rheological percolation threshold at *w_c_* = 1 wt.% (for a coefficient of correlation, R^2^, of 0.96). Hence, a complete agreement was observed between the electrical and rheological percolation thresholds. Pötschke et al. [55] reported similar values for electrical and rheological percolation thresholds (1 wt.% and 0.5 wt.%, respectively) for an MWCNT/polycarbonate composite. The fitting process also yielded a critical exponent of 1.74, which is slightly lower than the value obtained from the electrical conductivity data. Nevertheless, this value falls within the range reported for a three-dimensional network.

#### 3.1.3. Composites Morphology

**Figure 5:**
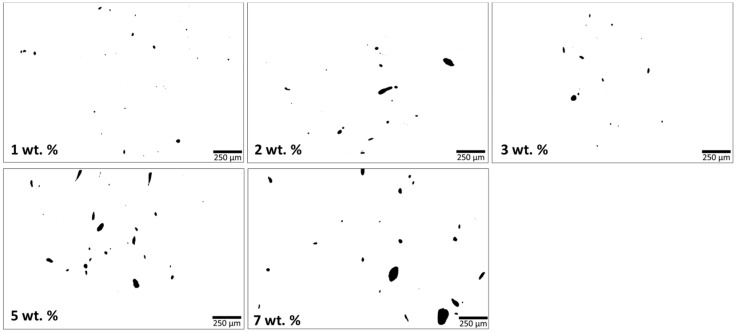
Optical micrographs of the cross-sections of MWCNT nanocomposites. The scale bar represents 250 μm.

The state of MWCNT agglomerates dispersion was evaluated by analysis of the nanocomposite micrographs obtained by transmission light microscopy of thin sections of PEEK/MWCNT extruded strands with 1 to 7 wt.% MWCNT, as illustrated in Figure 5. 

**Figure 6:**
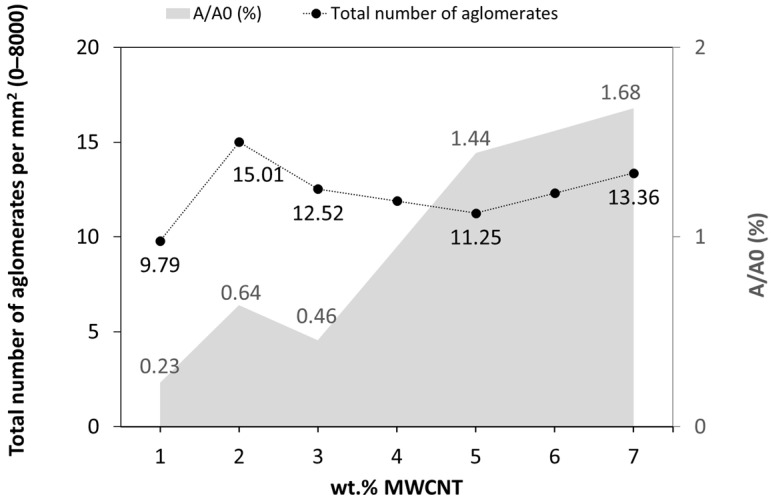
Total number of agglomerates per mm^2^ (classes 0–8000) and the agglomerate area ratio A/A0 as a function of the MWCNT wt.%.

Figure 5 depicts an increase in the agglomerates size and number with increasing MWCNT wt.%. Nanocomposites with 1 wt.% MWCNT exhibit good dispersion and distribution, with the smaller MWCNT agglomerates size. Figure 6 presents the number of agglomerates per unit area (mm^2^) and the agglomerate area ratio A/A0, for the nanocomposites with different MWCNT compositions. It is observed that the increase in MWCNT wt.% leads to the A/A0 increase. This is due to the high concentration of MWCNT and saturation of dispersion, reforming large agglomerates triggered by Van der Waals forces between the neighbouring MWCNT [56,57]. Socher et al. [24] studied different polymer/MWCNT composites, including the PEEK/1 wt.% MWCNT, reporting that an agglomerate area ratio < 0.1% represented good dispersion. The number of agglomerates per unit area remains reasonably constant for all MWCNT contents. This result is interesting, showing a uniform number of agglomerates formed during processing, that increase in size with the increase in MWCNT wt.%.

**Figure 7:**
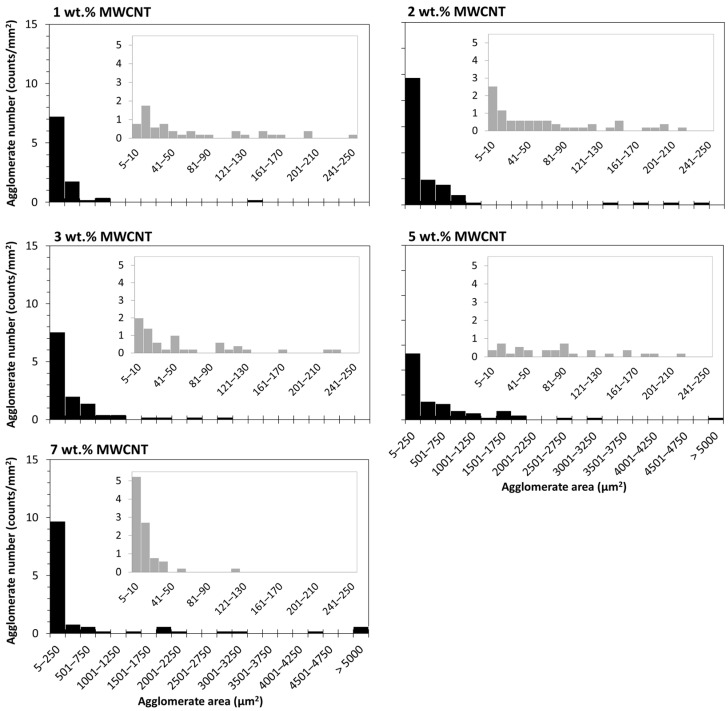
Distribution of the agglomerate areas for the nanocomposites with 1 to 7 wt.% MWCNT in the range of 5 to >5000 µm^2^. The inserts show the detailed area distribution for the smaller agglomerate sizes (5 to 250 µm^2^).

The size distribution of the agglomerates was quantitatively evaluated by image analysis of the optical micrographs. In Figure 7, the distribution of the agglomerate areas is presented against the number of agglomerates per unit area (mm^2^) for the nanocomposites with different MWCNT contents. These data are relevant since the presence of large agglomerates may potentially hinder the spinnability of the composites into the multifilament by leading to fibre breakage during the spinning process.

The analysis of Figure 7, representing the number of agglomerates with areas measured from 5 to >5000 µm^2^, shows that all nanocomposites present a greater number of agglomerates in the first class (5–250 µm^2^). The inserts depict the detailed distribution of the agglomerate sizes within the first class. This agglomerate size range is quite relevant for the later application in the melt-spinning of the multifilament. This effect was observed by Quijano [58], who reported fibre breakage during melt-spinning of PC/MWCNT, with an agglomerate area ratio > 0.73% (>3 wt.% MWCNT). Few studies reported that agglomerates having a size greater than the acceptable limit for the spinning process, between 1 and 10 μm (areas with approximately 1 and 100 μm^2^), may cause spinning breakages. Larger agglomerates should be filtered before passing the extruder die [38,39]. All composites present few agglomerates with areas greater than 100 μm^2^. Nevertheless, even at a residual amount, the larger agglomerates strongly contribute to the overall agglomerate area ratio. 

**Figure 8:**
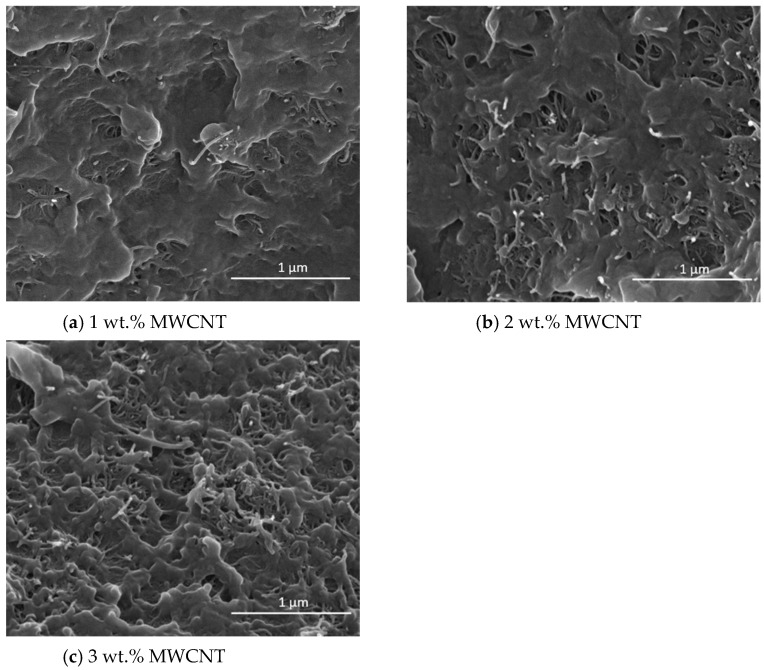
SEM micrographs of nanocomposites containing (**a**) 1, (**b**) 2, and (**c**) 3 wt.% MWCNT.

Figure 8 shows SEM images of composites’ fracture surfaces, within the electrical percolation threshold, containing 1, 2, and 3 wt.% MWCNT. The MWCNTs, which appear as bright spots, seem to be homogeneously dispersed within the PEEK matrix in both types of composites. All samples show a good interfacial adhesion between the filler and matrix phases.

#### 3.1.4. Thermal Stability

**Figure 9:**
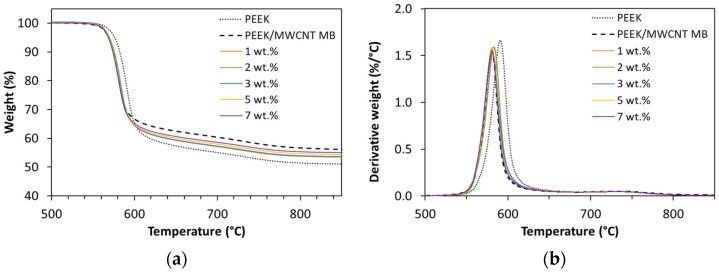
Thermogravimetric analysis, (**a**) TGA and (**b**) DTGA, of the neat PEEK, PEEK/MWCNT MB, and PEEK filled with 1 to 7 wt.% MWCNT (for comparative purposes, only the temperature range between 500 and 850 °C is plotted).

The thermal stability of the nanocomposites was studied by TGA under nitrogen atmosphere. The degradation curves of neat PEEK and nanocomposites containing 1, 2, 3, 5, and 7 wt.% MWCNTs, as well as the masterbatch (MB), are displayed in Figure 9. Thermal decomposition of neat PEEK is a two-step process. The first step is attributed to the random chain scission of ether and ketone bonds, and starts at ~570 °C, showing the maximum rate of weight loss (T_mr_) at ~590 °C. The second step occurs above 700 °C and is ascribed to the cracking and dehydrogenation of crosslinked residues produced in the first step. These processes result in a thermally stable carbonaceous char. Neat PEEK presents high amounts of char residues (~51 wt.%) after thermal degradation, consistent with other studies [28,59,60,61]. 

The MWCNT composites presented a slightly lower onset decomposition temperature than PEEK, that may be associated with the lower thermal stability of the MB, compared to the neat PEEK selected for MB dilution. The MB is formed by a different PEEK of unknown source. Otherwise, the addition of MWCNT to PEEK has been reported to induce a higher thermal stability compared to PEEK alone [1,62]. Table 2 presents the relevant temperatures, namely the temperature at which a reduction of 5 wt.% is observed by thermal degradation, T_d5_, the temperature of maximum weight loss rate, T_mr_, and the residual weight obtained at 850 °C.

### 3.2. Influence of the Extrusion Conditions 

In the present study, a PEEK/MWCNT masterbatch was used, and thus the primary agglomerates were pre-dispersed in a neat PEEK matrix. The influence of the extrusion conditions on the dispersion of the MWCNT in PEEK was studied for the composition with 5 wt.% MWCNT, obtained by masterbatch dilution, varying the extrusion parameters as described in Table 3. The 5 wt.% of MWCNT composition, well above the electrical percolation threshold, was selected, aimed at evaluating its possible future application in the melt-spinning process, producing electrically conductive melt-spun fibres [41,42]. The effect of the processing conditions upon the dispersion of the MWCNT in PEEK was assessed through the nanocomposite’s electrical conductivity, morphology, and the melt rheology. 

#### 3.2.1. Electrical Conductivity 

**Figure 10:**
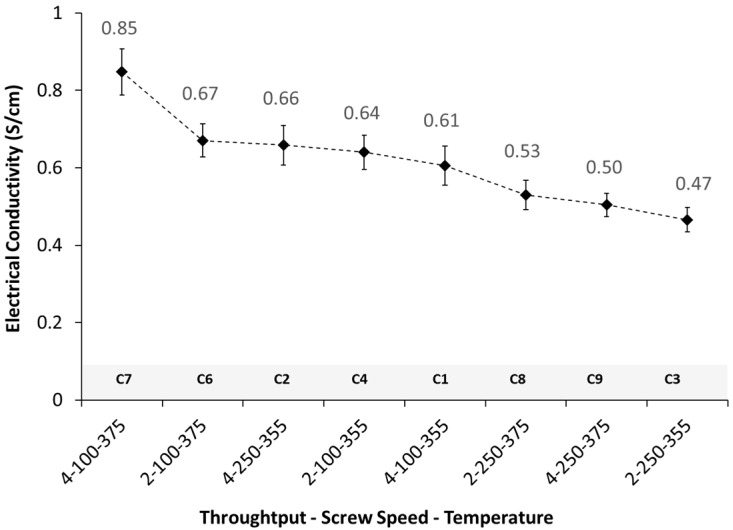
Electrical conductivity of PEEK/5 wt.% MWCNT nanocomposites prepared under varying processing conditions.

The electrical conductivity of the eight nanocomposites produced is presented in Figure 10. All the nanocomposites were electrically conductive, with conductivity values ranging from 0.47 to 0.85 S/cm. The nanocomposite with higher conductivity was C7, obtained at the higher temperature (375 °C) and throughput (4 kg/h), and at the lower screw speed (100 RPM).

In order to assess the effect of each process parameter on the composite’s morphology and rheology, the nanocomposite with the higher electrical conductivity value, C7, was selected as the reference material. To assess the effect of each process parameter individually, three other nanocomposites where chosen, each one varying only one processing parameter relative to C7. Table 4 summarizes the nanocomposites selected and processing parameters evaluated.

#### 3.2.2. Composites Morphology

The quality of the MWCNT dispersion was evaluated through the analysis of the morphology observed by optical microscopy. The agglomerate area ratio A/A0 and the distribution of the agglomerate areas were investigated for the composites with 5 wt.% MWCNT prepared under different processing conditions.

**Figure 11:**
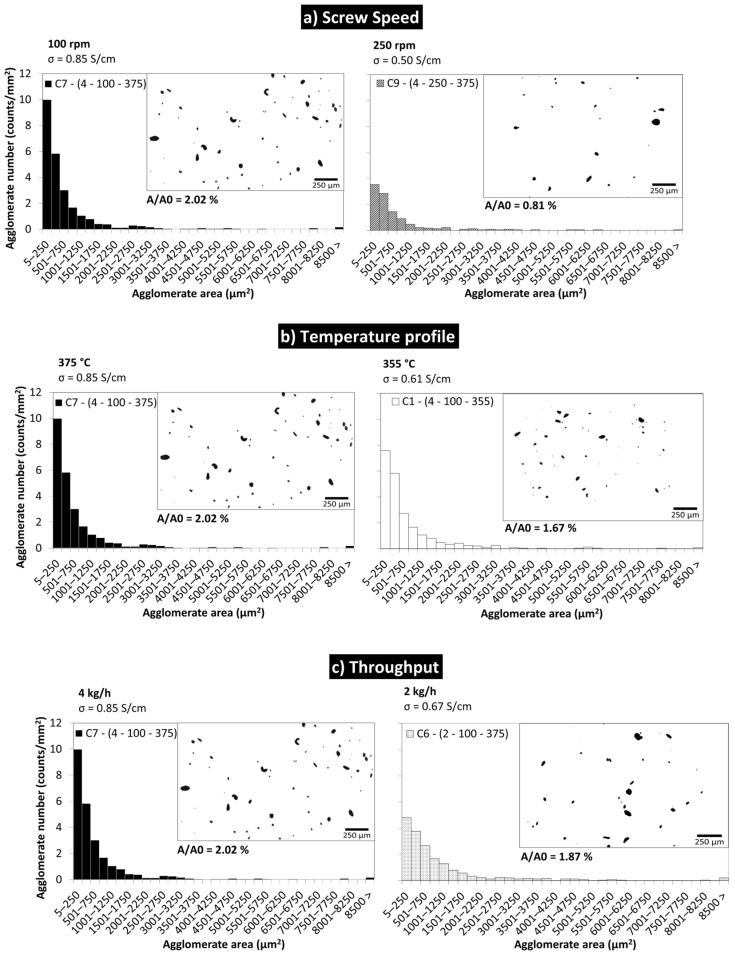
Influence of the extrusion parameters on the morphology (agglomerate area distribution), electrical conductivity, and agglomerate area ratio of PEEK/5 wt.% MWCNT nanocomposites.

Figure 11 depicts the effect of the selected process parameters on the morphology, electrical conductivity, and agglomerate area ratio of the nanocomposites. 

The increase in screw speed (Figure 11a) has a clear effect on the MWCNT dispersion, considerably reducing the A/A0 from 2.02 to 0.81%, and largely reducing the number of agglomerates per unit area. A decrease in electrical conductivity is observed; however, it is quite small, as the MWCNT wt.% is well above the percolation threshold. This is in line with the results reported by Krause et al. [12] and Pötschke et al. [32], that above the electrical percolation threshold, increasing screw speed leads to enhanced breakage of the MWCNT, decreasing the DC conductivity. Kasaliwal et al. [36] found a pronounced decrease in the size and number of agglomerates with the increase in screw speed, in line with the present work. However, the literature also reports the preparation of nanocomposites using higher screw speeds that lead to enhanced MWCNT dispersion and increased electrical conductivity [16,18,33,36,37,43]. In fact, during processing, several factors may affect the MWCNT morphology, dispersion, and composite properties in different directions. For example, (1) the occurrence of MWCNT breakage during melt-mixing is favoured at higher screw speeds and higher polymer viscosities, resulting in lower electrical conductivity; (2) low polymer viscosity and longer residence time may enhance polymer wrapping around well-dispersed nanotubes, leading to MWCNT insulation and thus lower electrical conductivity, even at the best MWCNT agglomerate dispersion; (3) higher screw speeds (thus shorter residence times) may not be efficient at disentangling primary MWCNT agglomerates, and may lead to agglomerate dispersion by breaking into small agglomerates but limiting the formation of an MWCNT conductive network. For all these reasons, it is recognized that the optimization of the MWCNT composites’ electrical conductivity requires adjusting the processing conditions, accounting for the polymer grade and its chemical, physical, and rheological characteristics. 

Figure 11b depicts the effect of temperature. An increase in the temperature profile from 355 to 375 °C led to a marginal increase in electrical conductivity, although larger agglomerates survive at higher processing temperatures. Tambe et al. [35] report that high processing temperatures dramatically decrease the electrical conductivity, associating this observation with the lower polymer viscosity that allows an extensive adsorption of polymer chains into the MWCNTs agglomerates and on the dispersed MWCNT surface. Kasaliwal et al. [37] observed that mixing at higher melting temperatures could form composites with low electrical resistivity and good agglomerates dispersion. Krause et al. [12] also report that the MWCNT dispersion was enhanced at higher melting temperatures, and the electrical conductivity increased due to the formation of an MWNT network in the polymer matrix. Similar results were reported by Hu et al. [63], who observed the formation of nanotube agglomerates at higher temperatures, which was found to be beneficial for the electrical conductivity of the nanocomposites. S. Pegel et al. [64] reported that the formation of secondary agglomerates of initially well-dispersed MWNT during processing can enhance the electrical conductivity. The present work corroborates these studies, as a small increase in the electrical conductivity was observed, associated with an increase in the agglomerates area ratio (from 1.67 to 2.02%).

Figure 11c compares the effect of throughput on the dispersion and agglomerate area ratio (A/A0) of PEEK/5 wt.% MWCNT nanocomposites, keeping the temperature profile and screw speed constant. The electrical conductivity slightly increased (0.67 to 0.86 S/cm) with the increase in throughput from 2 to 4 kg/h, although presenting a slightly higher agglomerate area ratio. However, the variation in conductivity and agglomerate morphology is marginal; thus, throughput has a small effect upon the nanocomposite’s properties. Villmow et al. [18] reported that an increase in throughput (i.e., the increase in flow rate) resulted in worse dispersion of MWCNT and lower electrical conductivity as a result of the shorter residence time, in particular when associated with low shear acting on the polymer melt [65]. However, the industrial production of PEEK/nanofiller composites would benefit from both high throughput and good dispersion of the nanofillers. 

**Figure 12:**
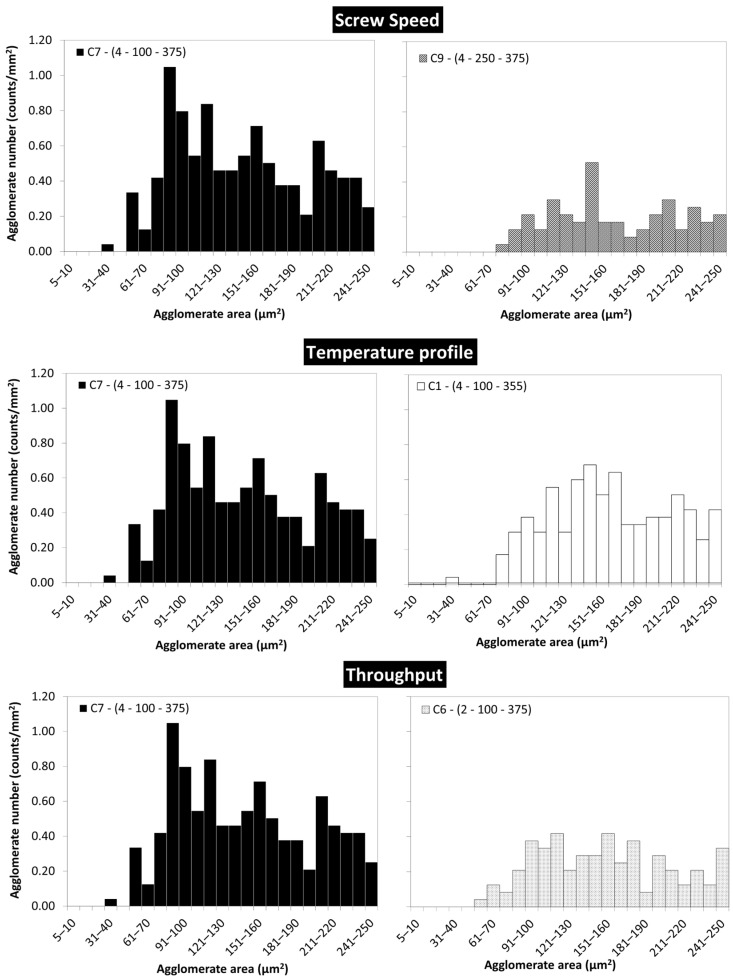
Zoom in on the class 5–250 µm^2^, presenting the macrodispersion of PEEK/5 wt.% MWCNT nanocomposites.

In order to produce filaments by melt-spinning, the agglomerates size should be as small as possible, ideally absent. Figure 12 depicts the agglomerate size distributions measured for PEEK/5 wt.% MWCNT nanocomposites produced under different processing conditions, focusing on the smaller agglomerates size range (5–250 µm^2^). A smaller number of agglomerates was observed for composites C1 and C6, and notably fewer for C9. 

It should be noted that composites with larger agglomerates than recommended for melt-spinning processing may still be used with this technology. Several authors [58,66,67] reported the production of multicomponent fibres by melt-spinning using composites containing larger agglomerates than recommended for melt-spinning, nevertheless affecting the fibres’ mechanical properties. 

#### 3.2.3. Rheological Analysis

**Figure 13:**
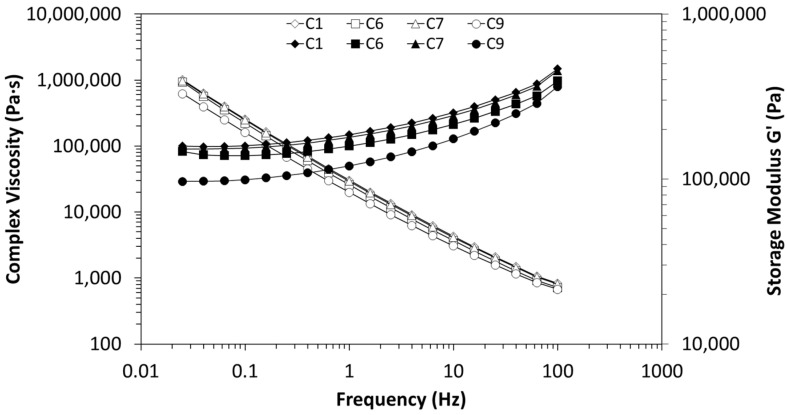
Frequency-dependent behaviour of the dynamic complex viscosity, as well as the storage modulus for the selected nanocomposites: C1, C6, C7, and C9.

The effect of the mentioned extrusion conditions (screw speed, throughput, and temperature) on the linear viscoelastic response, of the selected composites (C1, C6, C7, and C9, described in Table 3), at 400 °C, is depicted in Figure 13. After examining the frequency-dependent behaviour of the dynamic complex viscosity, it becomes evident that all nanocomposites demonstrate plastic behaviour across the entire frequency range, due to the high MWCNT content. It is also observed that the nanocomposite C9 stands out prominently from the others, displaying lower viscosity. This behaviour can be attributed to the fact that C9 was processed at a higher screw speed and temperature profile, leading to increased matrix degradation. The smaller number of agglomerates can, in fact, contribute to a lower viscosity (and conductivity) when comparing to the C7 nanocomposites, since the particle–particle distance is higher. Regarding the other nanocomposites (C1, C6, and C7), no significant differences were observed in complex viscosity. This may suggest that screw speed is the extrusion parameter with a higher impact on the nanocomposites processing, given that only C9 was processed at 250 rpm. With the other studied extrusion parameters, temperature profile and throughput, no significant differences were observed in the viscosity, which is in good agreement with the morphological and electrical analysis. Figure 13 also presents experimental results for the storage modulus as a function of frequency at 400 °C. For all nanocomposites, processed with different extrusion parameters, *G*′ reaches a plateau at low frequencies. It is well known that a plateau of *G*′ observed in the region of low frequency manifests the presence of a network or network-like structure formed in the melt [68], thus confirming the MWCNTs percolation. 

In line with the complex viscosity behaviour, the nanocomposite C9 exhibits the lowest value of elastic modulus. This behaviour can be attributed to both higher matrix degradation and lower connectivity between the agglomerates, resulting in reduced elasticity. With respect to the other nanocomposites C1, C6, and C7, no notable differences are observed in the storage modulus. Hence, the generated morphology in these nanocomposites is not significantly different, preventing variations in the elastic modulus.

## 4. Conclusions

PEEK is a high-performance thermoplastic suitable for demanding applications requiring high mechanical and thermal properties. It is an interesting material for technical textiles, and the addition of MWCNT could add new properties and enable new applications such as sensing. However, the literature is scarce concerning the production of PEEK nanocomposites, and even more limited concerning the production of PEEK multifilament’s. The present work contributes to the study of the effect of the processing conditions on the properties of PEEK/MWCNT nanocomposites, aiming at the production of electrically conductive composites minimizing the size and number of MWCNT agglomerates, that may be suitable for further melt-spinning of multifilament’s with application in high-performance textiles.

PEEK/MWCNT composites were extruded, and the MWCNT dispersion, electrical conductivity, and rheological properties were studied with the objective of maximizing the electrical conductivity while ensuring good MWCNT and rheological properties suitable for further production of melt-spun fibres (multifilament). The electrical percolation threshold of PEEK/MWCNT composites was found between 1 and 2 wt.% MWCNT. Electrical conductivity levels at the electrostatic dissipation and semiconductor range were attained for composites with 1 to 4 wt.% MWCNT. The composites with 5 wt% and higher MWCNT content can find application in electromagnetic shielding (>10^−2^ S/cm). To determine the rheologic percolation threshold, a power law slope (*n*) of plateau modulus was applied. The slope related to percolation was identified below the *ϕ_p_* < 1.5 wt.% for the PEEK/MWCNT nanocomposites, showing good correlation between the rheological and electrical percolation thresholds.

The morphology of MWCNT agglomerates and their dispersion, assessed by the agglomerate area distributions and agglomerate area ratio (A/A0), agreed with other studies, and showed a good dispersion level. The number of agglomerates per unit area remains reasonably constant for all MWCNT contents, showing a uniform number of agglomerates formed during processing that increase in size with the increase in MWCNT concentration. A few large agglomerates were found and may potentially hinder the spinnability of the composites into the multifilament by leading to fibre breakage during the spinning process. 

The addition of MWCNT resulted in a slightly lower onset decomposition temperature that may indicate a decrease in thermal stability of PEEK or may be associated to an increase in the thermal conductivity of the composites relative to neat PEEK. 

The influence of the more relevant extrusion process parameters on the dispersion and electrical properties of the composites shows small variations in MWCNT agglomerate dispersion and composite electrical conductivity with the variation in throughput, temperature profile, and screw speed, for PEEK/5 wt.% MWCNT nanocomposites. The major effect observed is associated with the increase in screw speed when the throughput and temperature profile are kept at the higher level. Under these conditions, the MWCNT agglomerate dispersion is considerably enhanced (A/A0 decreasing from 2.02 to 0.81%), at a negligible loss of electrical conductivity (from 0.85 to 0.50 S/cm). This result is of great importance for applications that require fine agglomerate dispersion, such as melt-spinning of multifilament’s. Due to the lower number and size of agglomerates, composite C9 can be the best fit for the melt-spinning process. 

Rheologic analyses shows that the nanocomposite C9 presented lower viscosity, possibly due to a matrix degradation (processed at higher temperature and screw speed). Screw speed was the parameter that revealed a higher impact.

## Figures and Tables

**Table 1:** Design of experiments for the PEEK/5 wt.% MWCNT nanocomposites.

Factors	Level		Unit
	Low	High	
Throughput	2	4	kg/h
Screw speed	100	250	RPM
Temperature profile	355	375	°C

**Table 2:** Temperature at 5% weight loss (T_d5%_), derivative weight maximum temperature (T_mr_), and residual weight of neat PEEK, PEEK/MWCNT MB, and nanocomposites.

Nanocomposites	T_d5%_ (°C)	T_mr_ (°C)	Residual Weight (%) at 850 °C
PEEK	574.50	590.89	51.09
PEEK/MWCNT MB	567.22	580.44	43.88
1% MWCNT	567.98	583.36	53.47
2% MWCNT	568.40	583.11	53.54
3% MWCNT	567.45	581.49	53.67
5% MWCNT	567.30	580.89	54.40
7% MWCNT	566.90	580.67	55.01

**Table 3:** Extrusion parameters selected for the preparation of the nanocomposites.

Throughput—Screw Speed—Temperature (kg/h—RPM—°C)	Composite Label
4-100-355	C1
4-250-355	C2
2-250-355	C3
2-100-355	C4
2-100-375	C6
4-100-375	C7
2-250-375	C8
4-250-375	C9

**Table 4:** Nanocomposites selected for the study of extrusion parameters.

Parameters Analysed	Composites Selected	Fixed Extrusion Parameters
Screw speed (100 vs. 250 RPM)	C7 vs. C9	Throughput (4 kg/h) and Temperature (375 °C)
Temperature (375 vs. 355 °C)	C7 vs. C1	Throughput (4 kg/h) and Screw speed (100 RPM)
Throughput (4 vs. 2 kg/h)	C7 vs. C6	Temperature (375 °C) and Screw speed (100 RPM)

## Data Availability

Data are contained within the article.
